# *Xenopsylla brasiliensis* Fleas in Plague Focus Areas, Madagascar

**DOI:** 10.3201/eid2212.160318

**Published:** 2016-12

**Authors:** Adélaïde Miarinjara, Christophe Rogier, Mireille Harimalala, Tojo R. Ramihangihajason, Sébastien Boyer

**Affiliations:** Université d'Antananarivo, Antananarivo, Madagascar (A. Miarinjara);; Institut Pasteur, Antananarivo (A. Miarinjara, C. Rogier, M. Harimalala, T.R. Ramihangihajason, S. Boyer);

**Keywords:** *Xenopsylla brasiliensis*, plague, flea, Madagascar, *Synopsyllus fonquerniei*, *Yersinia pestis*, *Pulex irritans*, *Xenopsylla cheopis*, vector-borne infections, zoonoses, bacteria

**To the Editor:** Plague is a life-threatening infectious disease caused by the gram-negative bacterium *Yersinia pestis* (*1*). *Y. pestis* primarily infects rodents but can also cause outbreaks of plague in humans. The infection is usually transmitted within murine populations and then to humans by bites from infected fleas. The oriental rat flea, *Xenopsylla cheopis*, is considered the most efficient plague vector (*1*). Plague remains a major public health threat, causing annual epidemics, especially in Madagascar.

From November 2013 through January 2014, Madagascar reported 427 suspected cases and 45 confirmed cases of plague (both bubonic and pneumonic) in 4 districts. We report here on the flea species associated with rodents and those collected from human dwellings in the Mandritsara District where plague occurred (Technical Appendix Figure). Four villages in the district were investigated 1 month after the end of the human plague epidemic and after an insecticide-based vector control intervention had taken place. Fleas were collected, either from rats or by using candle traps set inside houses, and preserved in 70% ethanol (online Technical Appendix Table). Rats were trapped alive inside houses and in the cultivated lands.

During the survey, 180 rodents were trapped; they belonged to species *Rattus rattus* (93.3%, n = 168), *Mus musculus* (5.6%, n = 10), and *Suncus murinus* (1.1%, n = 2). A total of 50 fleas were collected from these rodents. The fleas belonged to 4 species: *Sinopsyllus fonquerniei* (n = 26), *Xenopsylla brasiliensis* (n = 14), *X. cheopis* (n = 9), and *Echidnophaga gallinacea* (n = 1) (Table). The first 3 are known to be *Y. pestis* vectors. Of fleas caught in candle traps placed inside houses, ≈98% were the human flea *Pulex irritans*, whose role in plague outbreaks is unknown (*2**,**3*). 

**Table:** Number of fleas collected from rodents and by candle traps per species and per study site, Madagascar, 2013–2014

Source	Flea species	Beranimbo	Ambiamamy	Sahakondro	Antsiatsiaka*
*Rattus rattus* rat	*Synopsyllus fonquerniei*	21	1	4	0
	*Xenopsylla cheopis*	1	0	0	8
	*Xenopsylla brasiliensis*	0	0	7	7
	*Echidnophaga gallinacea*	0	1	0	0
Candle trap	*Tunga penetrans*	2	0	3	1
	*Pulex irritans*	0	0	0	138
	*Synopsyllus fonquerniei*	0	0	0	1

*Control village where no plague cases and no insecticide treatment occurred.

Although *X. cheopis* and *S. fonquerniei* fleas are common *Y. pestis* vectors in Madagascar (*1*), the major finding of this study was the discovery of *X. brasiliensis* fleas, which may be involved in plague transmission in Madagascar. Fleas were identified to the species under binocular magnification by using systematic keys (*4**,**5*). Each flea specimen was identified independently by 2 different technicians. The morphologic identification of *X. brasiliensis* (Baker, 1904) was also confirmed by Jean-Claude Beaucournu (*6*). Specimens of *X. brasiliensis* fleas identified in this study exhibit the morphologic characteristics of the species, which distinguish it from *X. cheopis* fleas, as follows: antepygidial bristle of male is marginal, inserted on the long pedestal, process 1 of the clasper with 8 or 9 bristles (which are stout, straight, spiniform, and 1 angled) and the process 2 of the clasper with the tip turned up (*5*). Compared with females of other *Xenopsylla* spp., *X. brasiliensis* females have a distinct spermathecal shape with a very swollen bulga, which is larger than the base of the hilla (*4*). Moreover, DNA of *X. brasiliensis, P. irritans,* and *X. cheopis* fleas collected during this study was extracted and amplified by using primers targeting the D3 segment of the 28S ribosomal RNA-encoding gene (*7*) and sequenced. *X. brasiliensis* sequences isolated showed 100% nucleotide similarity with those from Mauritius (*4*) and were different from *X. cheopis* and *P. irritans* sequences. All sequences are available in GenBank (accession nos. KU759935–KU759954).

Given the vital maritime exchange between Madagascar, the countries of East Africa, and the islands of the Indian Ocean, the presence of *X. brasiliensis* fleas in Madagascar was almost predictable. *X. brasiliensis* fleas originated in sub-Saharan Africa and have spread to other parts of the world, notably Brazil and India (*8*). This species is among the most common flea species found on rodents in southern and eastern Africa, where it is considered a key *Y. pestis* vector, especially in rural environments (*9*). This species has been described on the Comoros archipelago and Mauritius since the early 20th century (*5*) and, more recently, on Reunion Island (*10*). However, to our knowledge, *X. brasiliensis* fleas had not previously been found in Madagascar, although >40 species of fleas have been identified in this country since the 1930s. In this study, we found that *X. brasiliensis* fleas were parasitizing *R. rattus* rats caught inside human dwellings. *R. rattus* rats are considered the main plague reservoir in Madagascar (*1*).

This study’s key finding is the discovery of a third vector species that may be involved in *Y. pestis* transmission in Madagascar. Further genetic studies are necessary to clarify when *X. brasiliensis* fleas arrived in Madagascar and where they originated. Additional studies are also needed to determine the distribution of *X. brasiliensis* fleas on the island and their role in plague transmission.

Technical Appendix Number of rodents and fleas trapped per house and per study site and location of study sites for plague vectors, Madagascar, 2013–2014.
