# Supplementary material for: Xenopsylla brasiliensis Fleas in Plague Focus Areas, Madagascar
Source: Emerg Infect Dis. 2016 Dec;22(12):2207–8. doi: 10.3201/eid2212.160318 (PMC5189135; doi:10.3201/eid2212.160318)
Supplement: Technical Appendix — Number of rodents and fleas trapped per house and per study site and location of study sites for plague vectors, Madagascar, 2013–2014. [file 16-0318-Techapp-s1.pdf]

Article DOI: http://dx.doi.org/10.3201/eid2212.160318

Xenopsylla brasiliensis Fleas in Plague Focus Areas, Madagascar

Technical Appendix

Technical Appendix Table. Number of rodents and fleas trapped per house and per study site, Madagascar, 2013–2014

	Rodents trapping		Candle traps	
Study sites	No. sampled houses	No. rodents	No. sampled houses	No. fleas
Beranimbo*	93	44	14	2
Ambiamamy	49	23	6	0
Sahakondro	113	94	20	3
Antsiatsiaka	97	19	19	140

*The only study site where outdoor trapping was done: 16 rodents were trapped.

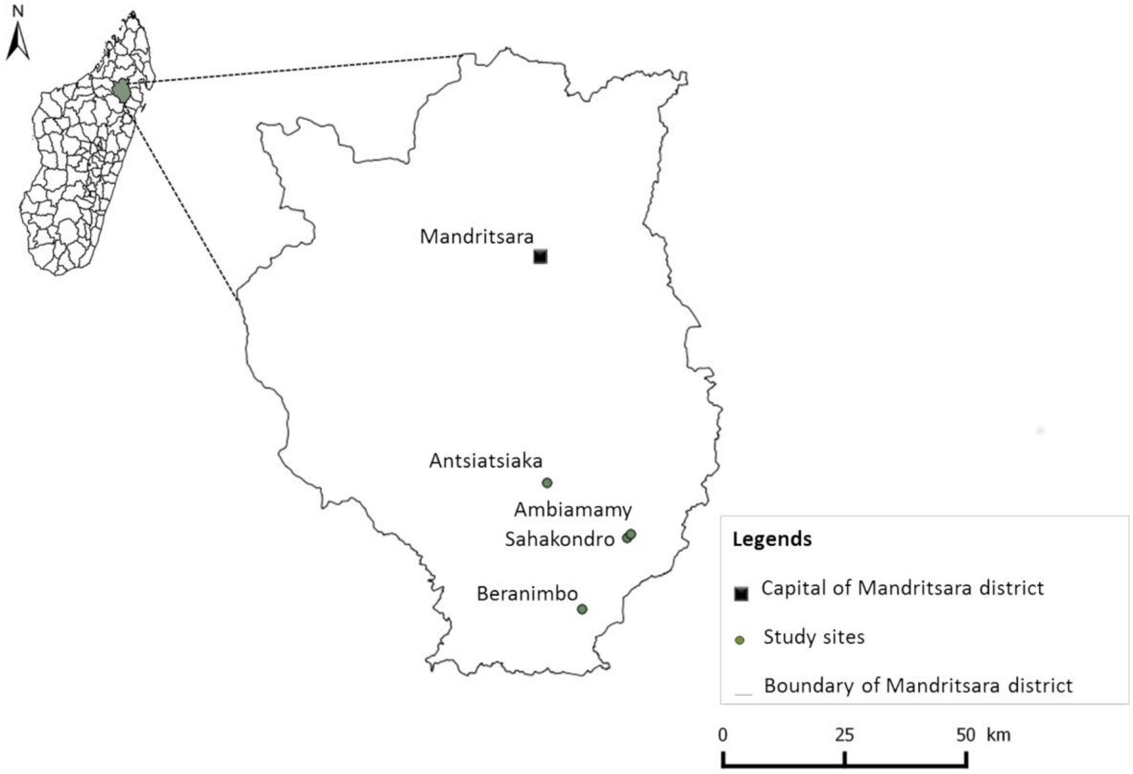

Technical Appendix Figure. Location of study sites for plague vectors, Madagascar, 2013–2014.
